# Tobacco Cessation, Rural Residence, and Lung Cancer

**Published:** 2020-02-17

**Authors:** Steven S. Coughlin, Marlo Vernon, Ban Majeed, Catherine Clary, Justin Moore, K.M. Islam, Martha S. Tingen

**Affiliations:** 1 Department of Population Health Sciences, Medical College of Georgia, Augusta University, Augusta, GA; 2 Institute of Public and Preventive Health, Augusta University, Augusta, GA; 3 Department of Medicine, Medical College of Georgia, Augusta University, Augusta, GA; 4 Georgia Cancer Center, Augusta University, Augusta, GA

## Introduction

Tobacco use remains the leading cause of preventable mortality and morbidity in the U.S., responsible for nearly 443,000 deaths annually (Fu et al., 2014; CDC, 2011). Cigarette smoking has been causally linked to numerous types of cancer (lung, mouth, nasal cavity, throat, laryngeal, esophageal, stomach, colon, liver, pancreatic, bladder, cervical, acute myeloid leukemia); cardiovascular disease (heart disease, stroke, aortic aneurysm); diabetes; rheumatoid arthritis; age-related macular degeneration; and respiratory illness (chronic bronchitis, emphysema) (NCI 2014). Smoking also contributes to respiratory infections (e.g., pneumonia) and, if a mother smokes while pregnant, to low birth weight and premature birth, the primary causes of infant mortality. Use of other tobacco products such as cigars or pipes and electronic cigarettes also increase the risk of cancer. In the U.S., cigarette smoking causes about 90% of lung cancers. According to the Centers for Disease Control and Prevention (CDC), tobacco smoke is a toxic mixture of more than 7,000 chemicals, of which at least 70 are known to cause cancer. In addition to nicotine, tobacco users are exposed to several classes of carcinogenic and other toxicants such as tobacco-specific nitrosamines (TSNAs), volatile organic compounds (VOC), and polycyclic aromatic hydrocarbons (PAHs), all of which have been linked to cancer, heart and lung diseases (U.S. Department of Health and Human Services, 2010). Individuals who smoke are 15–30 times more likely to develop lung cancer and die from lung cancer than people who do not smoke (NCI 2014).

Most cigarette smokers want to quit smoking, and about 50% make a quit attempt each year, but only 6% achieve long-term cessation (CDC 2011). Effective treatments exist, and the evidence-based clinical practice guideline recommends a combination of behavioral skills counseling and pharmacother apy because this multi-modality approach is most effective for helping smokers quit over the long-term (Fiore et al., 2008). Since their introduction in the mid-2000s, e-cigarettes have been advertised as an effective nicotine delivery system for current smokers seeking alternative cessation strategies; however, there is no substantiated research that confirms this. As of February 4, 2020, 64 deaths have resulted from e-cigarette use and an additional 2,694 hospitalizations from e-cigarette, or vaping, product use-associated lung injury (EVALI), a new diagnosis for e-cigarette associated illnesses. (CDC citation below) Of those hospitalized or who have died, 37% were 18 – 24 years of age. This is representative of the fact that e-cigarette use is highest among youth and young adults. Also, concurrent (combustible plus e-cigarette) users have estimates ranging from 8.2% to 10.6% (Jaber et al., 2018; Levy, Yuan, & Li, 2017; Mirbolouk et al., 2018). Although traditional tobacco-control efforts have proven beneficial for many populations, vulnerable subpopulations need further interventions. One plausible approach is to expand effective cessation strategies to those of disadvantaged sociodemographic backgrounds.

The U.S. Public Health Service Clinical Practice Guideline for the Treatment of Tobacco Dependence provides best practice standards for treating tobacco dependence (Fiore et al. 2008). Techniques stemming from behaviorally based counseling models, including motivational enhancement and skills training, are effective for tobaccocessation (Gritz et al. 2006). The provision of social support is also beneficial. Pharmacotherapies for tobaccocessation include nicotine replacement therapy (nicotine patch, gum, inhaler, spray, and lozenge) the antidepressant, bupropion, and the newest agent Varenicline (Chantix). A variety of evidence-based public health and clinical interventions are available to help people quit smoking, as systematically reviewed by the Guide to Community Preventive Services (http://www.thecommunityguide.org/index.html) and by the U.S. Preventive Services Taskforce (http://www.uspreventiveservicestaskforce.org/recommendations.htm).

Despite the high prevalence of interest in quitting and quit attempts without effective treatments, our health care system approach to tobacco cessation is predominately reactive. Smokers and e-cigarettes users either have to request treatment or have a clinical encounter in which their provider has the initiative, time, and capacity to either refer to tobacco cessation care or offer and deliver in-office tobacco cessation care. A proactive tobacco cessation care model with low barriers to access may greatly increase the reach of evidence-based treatment (Fu et al., 2014). Contacting all smokers as a matter of concern and offering help with quitting is a proactive approach to cessation (as compared with the more common, reactive approach) (Danan et al., 2018).

Tobacco-cessation counseling tends to preferentially benefit high-socioeconomic status (SES) smokers, potentially exacerbating disparities (Danan et al., 2018; Haas et al., 2015). Interventions that include proactive outreach, counseling, and free or low-cost cessation counseling and pharmacotherapy will be more feasible for helping low-SES smokers quit (Danan et al., 2018.

## Rural Residents and Tobacco Cessation

Although the feasibility, acceptability, and effectiveness of proactive approaches to tobaccocessation have been examined in randomized controlled trials (RCTs) (Fu et al., 2014; Fu et al., 2016; Danan et al., 2018; Haas et al., 2015; Rogers et al. 2018; Sherman et al., 2018), including trials aimed at low-income patients and patients seen in primary care, tobacco cessation interventions for rural residents (including those newly diagnosed with lung cancer) have been tested minimally in RCTs (Caldwell, Tingen et al, 2018).

Residents of rural areas are more likely to be low-income and to lack health insurance than residents of urban areas. Cigarette smoking rates are higher in socioeconomically disadvantaged populations than in more affluent populations (Fu et al., 2016). In the U.S., 28% of adults living below the federal poverty line smoke cigarettes compared with 17% of adults at or above the poverty level (Ahmed et al., 2014). Smoking cigarettes accounts for up to half the mortality difference between low- and high-SES men and women (Danan et al., 2018). Adults who believe that e-cigarettes are less addictive than tobacco cigarettes were 2.5 more likely to try e-cigarettes. Using the HINTS-Food and Drug Administration data, recent reports indicate that rural and urban respondents have a similar history of e-cigarette use, but that rural respondents were significantly more likely to trust faith-based leaders and tobacco companies when seeking information about e-cigarettes (Lewis-Thames et al., 2020). Among youth who had never smoked, e-cigarette use was strongly associated with cigarette initiation (Kintz et al., 2020). Among adults younger than 65 years, 16% of those with private health insurance are current smokers, compared with 34% of Medicaid recipients and 32% of the uninsured (Schiller et al., 2010).

Socioeconomically disadvantaged smokers, such as those who reside in rural areas, are less likely to use and have access to evidence-based tobacco cessation treatments (pharmacotherapy and behavioral skills counseling) than the general population of smokers (Fu et al., 2014; Cokkinides et al., 2005). Factors that contribute to the disparity in evidence-based treatment use include greater life stressors that reduce motivation to quit, lack of knowledge about the benefits of using pharmacotherapy and lack of awareness regarding Medicaid coverage for smoking cessation treatment (McMenamin et al., 2004). Other barriers include a lower likelihood of receiving preventive care services, difficulty taking time from work for cessation services, travel time and an inability to pay out-of-pocket expenses for pharmacotherapy (Blumenthal, et al. 2007).

A substantial body of evidence indicates that quit lines for cessation are effective in helping people to stop smoking; however, there is a paucity of research on quit line’s effectiveness in rural populations. Also, the relative effectiveness of quit lines to smoking cessation when provided by oncology nurses is unknown.

## Lung Cancer and Smoking Cessation

Lung cancer is the leading cause of cancer-related death for men and women in the United States, with annual mortality exceeding that for breast, prostate, colon, and pancreatic cancers combined (Jenkins et al., 2018). Of particular concern is the rural-urban disparities in lung cancer incidence and mortality. Reports show slower reduction in cancer death rates in rural America compared with urban areas and higher rates of new cases and death rates for lung cancer in rural America.(Redfield et al., 2008)

Although lung cancer incidence, late stage incidence, and mortality rates are elevated in rural areas, recent advances such as the use of low-dose CT for lung cancer screening have lagged behind in rural areas (Jenkins et al., 2018). Recent updates to the USPTF Lung Cancer screening guidelines recommend a low-dose CT (LDCT) scan as the most effective screening tool (Moyer, 2014). A previous national evaluation found that less than 5% of eligible Medicare patients received this screening, even though Medicare extended coverage to eligible patients(Nishi, Zhou, Kuo, & Goodwin, 2019). Rural populations may experience greater system, provider, and individual-level barriers to health-seeking behaviors (e.g., tobacco cessation) (Jenkins et al., 2018). The frequency of smoking at the time of diagnosis of lung cancer is estimated to be 37% (Cooley et al., 2009).

Unlike risk factors such as family history and radiation exposure, smoking is a modifiable risk factor that not only increases the risk for lung cancer and other cancers, yet also adversely affects the outcomes of cancer treatment(Siegel, Miller, & Jemal, 2019). Cigarette smoking is harmful for cancer patients and cancer survivors in several ways (Gallaway et al., 2019). For cancer patients who are current smokers at the time of diagnosis, entering smoking cessation significantly improves cancer treatment success and reduces risk of dying by 30–40% (Gritz, Toll, & Warren, 2014; National Center for Chronic Disease Prevention and Health Promotion (US) Office on Smoking and Health, 2014). Continued smoking and tobacco use during cancer treatment can result in increased risk of cancer recurrence and poorer treatment responses (Cinciripini et al., 2019). Continued smoking after a cancer diagnosis is associated with adverse health outcomes, including increased all-cause mortality and increased cancer-specific mortality, negative effects on cancer treatment and worsened chemotherapy toxicity, and increased risk for a second malignancy at the same site or a different site (Gallaway et al., 2019). Oncologists and other physicians have reported negative perceptions of their patients’ willingness to quit and are not typically trained in health behavior modification interventions (Weaver et al., 2012). A previous intervention providing tobacco cessation to cancer patients reported high and sustained quit rates. (Cinciripini et al., 2019). Quitting smoking after a cancer diagnosis can improve a patient’s cancer prognosis and increase long-term survival (HHS 2014). Leading cancer organizations including the American Society for Clinical Oncology, the American Association for Cancer Research, the Oncology Nursing Society, the International Association for the Study of Lung Cancer, and the National Comprehensive Cancer Network have advocated for providing cessation support to cancer patients (Warren et al., 2015).

The feasibility, acceptability, and effectiveness of proactive approaches to smoking and tobacco cessation have been examined in randomized controlled trials (RCTs)(Fu et al., 2014; Fu et al., 2016; Danan et al., 2018; Haas et al., 2015; Rogers et al. 2018; Sherman et al., 2018), although the effectiveness of smoking cessation programs for lung cancer patients has not previously been studies in rural populations where barriers to tobacco cessation services are potentially greater than in urban areas.

Studies involving lung cancer patients in rural areas are needed to compare the relative effectiveness of interventions such as smoking quit lines and tobaccocessation counseling provided by nurse navigators or trained cessation educators. More scientific evidence is needed to address questions that affect outcomes of interest to lung cancer patients and their caregivers.

## References

[R1] Ahmed J, Agaku IT, O’Connor, Current cigarette smoking among adults—United States, 2005–2014. (2015) MMWR Morb Mortal Wkly Rep 64(44):1233–1240. 26562061 10.15585/mmwr.mm6444a2

[R2] Blumenthal DS Barriers to the provision of smoking cessation services reported by clinicians in underserved communities. (2007) J Am board Fam Med 20(3): 272–279 17478660 10.3122/jabfm.2007.03.060115

[R3] Caldwell AL, Tingen MS, Nguyen JT, Parental smoking cessation: Impacting children’s tobacco smoke exposure in the home. (2018) Pediatrics 141(Suppl 1): S96–S106. 29292310 10.1542/peds.2017-1026M PMC5745674

[R4] Centers for Disease Control and Prevention (CDC). Vital signs. (2011) MMWR Morb Mortal Wkly Rep 60:1207–1212. 21900875

[R5] Centers for Disease Control and Prevention (CDC). Quitting smoking among adults. (2011) MMWR Morb Mortal Wkly Rep 60(44):1513–1519. 22071589

[R6] Centers for Disease Control and Prevention. Outbreak of Lung Injury Associated with the Use of E-Cigarette, or Va-ping, Products. Accessed on February 12, 2020

[R7] Cinciripini PM, Karam-Hage M, Kypriotakis G, Association of a comprehensive smoking cessation pro gram with smoking abstinence among patients with cancer. (2019) JAMA Netw Open 2(9): e1912251–e1912251. 31560387 10.1001/jamanetworkopen.2019.12251 PMC6777393

[R8] Cokkinides VE, Ward E, Jemal, Under-use of smoking-cessation treatments: results from the National Health Interview Survey, 2000. (2005) Am J Prev Med 28(1):119–122. 15626567 10.1016/j.amepre.2004.09.007

[R9] Cooley ME, Lundin R, Murray L Smoking cessation interventions in cancer care: opportunities for oncology nurses and nurse scientists. (2009) Annu Rev Nurs Res 27: 243–272. 20192107 10.1891/0739-6686.27.243 PMC4729378

[R10] Cummings KM, Dresler CM, Field JK, E-cigarettes and cancer patients. (2014) J Thorac Oncol 9(4): 438–441. 24736063 10.1097/JTO.0000000000000129 PMC4040965

[R11] Danan ER, Fu SS, Clothier BA, The equity impact of proactive outreach to smokers: analysis of a randomized trial. (2018) Am J Prev Med 55(4): 506–516. 30139707 10.1016/j.amepre.2018.05.023

[R12] Fiore MC, Jaen CR, Baker TB, Clinical Practice Guideline. Rockville, MD: U.S. Department of Health and Human Services, Public health Service; 2009. Treating tobacco use and dependence: 2008 update.

[R13] Fu SS, van Ryn M, Sherman SE, Proactive tobacco treatment and population-level cessation. A pragmatic randomized controlled trial. (2014) JAMA Intern Med 174(5): 671–677. 24615217 10.1001/jamainternmed.2014.177

[R14] Fu SS, van Ryn M, Nelson D, Proactive tobacco treatment offering free nicotine replacement therapy and telephone counselling for socioeconomically disadvantaged smokers: a randomized clinical trial. (2016) Thorax 71(5): 446–453. 26931362 10.1136/thoraxjnl-2015-207904 PMC4862067

[R15] Gallay MS, Glover-Kudon R, Momin B, Smoking cessation attitudes and practices among cancer survivors—United States, 2015. (2019) J Cancer Surviv 13(1): 66–74. 30612253 10.1007/s11764-018-0728-2 PMC6387634

[R16] Gritz ER, Toll BA, Warren GW Tobacco use in the oncology setting: advancing clinical practice and research. (2014) Cancer Epidemiol Prev Biomarkers 23(1): 3–9. 10.1158/1055-9965.EPI-13-0896 PMC3893715 24420982

[R17] Gritz ER, Fingeret MC, Vidrine DJ, Successes and failures of the teachable moment. Smoking cessation in cancer patients. (2006) Cancer 106(1):17–27. 16311986 10.1002/cncr.21598

[R18] Haas JS, Linder JA, Park ER, Proactive tobacco cessation outreach to smokers of low socioeconomic status. A randomized controlled trial. (2015) JAMA Intern Med 175(2): 218–226. 25506771 10.1001/jamainternmed.2014.6674 PMC4590783

[R19] Jaber RM, Mirbolouk M, DeFilippis AP, Electronic Cigarette Use Prevalence, Associated Factors, and Pattern by Cigarette Smoking Status in the United States From NHANES (National Health and Nutrition Examination Survey) 2013–2014. (2018) J Am Heart Assoc 7(14): e008178. 30007934 10.1161/JAHA.117.008178 PMC6064855

[R20] Jenkins WD, Matthews AK, Bailey A, Rural areas are disproportionately impacted by smoking and lung cancer. (2018) Prev Med Reports 10: 200–203. 10.1016/j.pmedr.2018.03.011 PMC5984228 29868368

[R21] Kintz N, Liu M, Chou CP, Risk factors associated with subsequent initiation of cigarettes and e-cigarettes in adolescence: A structural equation modeling approach. (2020) Drug Alcohol Depend 207: 107676. 31816488 10.1016/j.drugalcdep.2019.107676 PMC6980983

[R22] Levy DT, Yuan Z, Li Y The Prevalence and Characteristics of E-Cigarette Users in the U.S. (2017) Int J Environ Res Public Health 14(10): pii: E1200. 29019917 10.3390/ijerph14101200 PMC5664701

[R23] Mirbolouk M, Charkhch P, Kianoush S, Prevalence and Distribution of E-Cigarette Use Among U.S. Adults: Behavioral Risk Factor Surveillance System, 2016. (2018) Ann Intern Med 169(7): 429–438. 30167658 10.7326/M17-3440 PMC10534294

[R24] Moyer VA Screening for lung cancer: US Preventive Services Task Force recommendation statement. (2014) Ann Intern Med 160(5): 330–338. 24378917 10.7326/M13-2771

[R25] National Cancer Institute. Harms of cigarette smoking and health benefits of quitting. (2014) [Accessed February 6, 2020]

[R26] National Center for Chronic Disease Prevention and Health Promotion (US) Office on Smoking and Health. (2014). The health consequences of smoking—50 years of progress: a report of the surgeon general. 24455788

[R27] Nish S, Zhou J, Kuo Y-F, Use of Lung Cancer Screening With Low-Dose Computed Tomography in the Medicare Population. (2019) Mayo Clin Proc Innov Qual Outcomes 3(1):70–77. 30899910 10.1016/j.mayocpiqo.2018.12.003 PMC6410337

[R28] Redfield RR, Bunnell R, Ellis B, Morbidity and Mortality Weekly Report Centers for Disease Control and Prevention MMWR Editorial and Production Staff (Weekly) MMWR Editorial Board. (2008)

[R29] Rogers ES, Fu SS, Krebs P, Proactive tobacco treatment for smokers using Veterans Administration Mental Health Clinics. (2018) Am J Prev Med 54(5): 620–629. 29551324 10.1016/j.amepre.2018.02.011

[R30] Schiller JS, Lucas JW, Ward BW, Summary health statistics for U.S. adults: National Health Interview Survey, 2011. (2012) Vital Health Stat 10 256: 1–218. 25116400

[R31] Sherman SE, Krebs P, York LS, Telephone care co-ordination for tobacco cessation: randomized trials testing proactive versus reactive models. (2018) Tob Control 27(1): 78–82. 28190003 10.1136/tobaccocontrol-2016-053327

[R32] Siegel RL, Miller KD, Jemal A Cancer statistics, 2019. (2019) CA Cancer J Clin 69(1): 7–34. 30620402 10.3322/caac.21551

[R33] US Department of Health and Human Services. The health consequences of smoking: 50 years of progress: a report of the surgeon general. Atlanta: Department of Health and Human Services, Centers for Disease Control and Prevention, national Center for Chronic Disease Prevention and Health Promotion, Office on Smoking and Health; 2014.

[R34] Warren GW, Dibaj S, Hutson A, Identifying targeted strategies to improve smoking cessation support for cancer patients. (2015) J Thoracic Oncol 10(11):1532–1537. 10.1097/JTO.0000000000000659 26317914

[R35] Weaver KE, Danhauer SC, Tooze JA, Smoking cessation counseling beliefs and behaviors of outpatient oncology providers. (2012) The Oncologist 17(3): 455–462. 22334454 10.1634/theoncologist.2011-0350 PMC3316932

